# Infective Endocarditis: Predictive Factors for Diagnosis and Mortality in Surgically Treated Patients

**DOI:** 10.3390/jcdd9120467

**Published:** 2022-12-19

**Authors:** Jing Li, Tamara Ruegamer, Christoph Brochhausen, Karin Menhart, Andreas Hiergeist, Lukas Kraemer, Dirk Hellwig, Lars S. Maier, Christof Schmid, Jonathan Jantsch, Christian Schach

**Affiliations:** 1 Department for Cardiac, Thoracic and Cardiovascular Surgery, University Heart Center Regensburg, Franz-Josef-Strauß-Allee 11, 93053 Regensburg, Germany; 2 Institute of Clinical Microbiology, University Hospital Regensburg, Franz-Josef-Strauß-Allee 11, 93053 Regensburg, Germany; 3 Department for Pathology, University Hospital Regensburg, Franz-Josef-Strauß-Allee 11, 93053 Regensburg, Germany; 4 Department for Nuclear Medicine, University Heart Center, Franz-Josef-Strauß-Allee 11, 93053 Regensburg, Germany; 5 Department for Internal Medicine II, University Heart Center Regensburg, Franz-Josef-Strauß-Allee 11, 93053 Regensburg, Germany; 6 Institute for Medical Microbiology, Immunology and Hygiene, University of Cologne, Goldenfelsstraße 19-21, 50935 Köln, Germany

**Keywords:** infective endocarditis, mortality, valve histopathology, NT-proBNP, ROC analysis, sensitivity, specificity

## Abstract

Background: Diagnosis of infective endocarditis (IE) often is challenging, and mortality is high in such patients. Our goal was to characterize common diagnostic tools to enable a rapid and accurate diagnosis and to correlate these tools with mortality outcomes. Methods: Because of the possibility of including perioperative diagnostics, only surgically treated patients with suspected left-sided IE were included in this retrospective, monocentric study. A clinical committee confirmed the diagnosis of IE. Results: 201 consecutive patients (age 64 ± 13 years, 74% male) were finally diagnosed with IE, and 14 patients turned out IE-negative. Preoperative tests with the highest sensitivity for IE were positive blood cultures (89.0%) and transesophageal echocardiography (87.5%). In receiver operating characteristics, vegetation size revealed high predictive power for IE (AUC 0.800, *p* < 0.001) with an optimal cut-off value of 11.5 mm. Systemic embolism was associated with mortality, and N-terminal prohormone of B-type natriuretic peptide (NT-proBNP) had predictive power for mortality. Conclusion: If diagnostic standard tools remain inconclusive, we suggest employing novel cut-off values to increase diagnostic accuracy and accelerate diagnosis. Patients with embolism or elevated NT-proBNP deserve a closer follow-up.

## 1. Introduction

Patients with infective endocarditis (IE) profit from early therapy [1,2]. Particularly left-sided IE has a relevant impact on morbidity and mortality (within 1 year up to 50%) [3], particularly if complications such as heart failure or systemic embolism (up to 80%) occur [4,5,6]. The modified Duke criteria are recommended by actual guidelines and are the most applied indicator for the diagnosis of IE [7,8,9]. These have an overall sensitivity of 80–90%, which is significantly lower in specific cases [10,11]. Especially in patients with prosthetic valves, the use of ^18^F-fluorodeoxyglucose positron emission tomography/computed tomography (F-18-FDG-PET/CT) may be helpful [12]. The valve vegetation is the nidus of IE and consequently serves as the main target for preoperative imaging and intraoperative diagnosis. According to international guidelines, histopathological (HP) examination of resected valvular tissue or embolic fragments remains the gold standard for the diagnosis of IE as it allows visualization of microorganisms and/or neutrophilic infiltrates [7,13]. In the case of echocardiographic evaluation, a vegetation size of >30 mm as a singular criterion indicates urgent surgical treatment, and a size > 10 mm was associated with increased mortality if not treated surgically [7,9,14]. It remains unclear if every patient with echocardiographic who detected “vegetations”—especially of small size—turns out to have found endocarditic lesions that are the best predictive parameters for diagnostic and therapeutic decision-making.

In this study, we investigated a surgically treated population with left-sided IE (which has a higher impact on morbidity and mortality compared to the right-sided IE) [6], which was diagnosed definitely by the modified Duke criteria and compared various pre- or perioperative diagnostic tools with the outcomes IE diagnosis, 30-day and 1-year mortality.

## 2. Materials and Methods

Study design, patient population and approval: In this single-center study, we screened retrospectively for patients with left-sided IE who underwent heart surgery between January 2015 and January 2020 at University Hospital Regensburg, Germany. Parameters were extracted from SAP (ERP 6.0, Walldorf, Germany) or Swisslab (Roche Diagnostics IT Solutions, Berlin, Nexus AG, Berlin, Germany) and then collected in a Microsoft Excel spreadsheet in a pseudonymized manner (Microsoft Excel 2019, Redmond, DC, USA). The study was approved by the institutional review board of the University Regensburg, Germany (approval number 20-1911-104).

Surgical indications and procedure: Indications for surgery were established according to the European EACTS/ESC guidelines [7]. Open-heart surgery for IE was performed with a standard heart–lung machine and via median sternotomy. Surgical principles included radical debridement of all infected tissues, opening and cleaning of all abscess cavities and necrotic fistulas, and finally, valve replacement.

Clinical laboratory analyses: All clinical laboratory analyses were carried out from fresh peripheral venous blood samples at admission. The determination of C-reactive protein (CRP), procalcitonin (PCT), and NT-proBNP was performed according to standard procedures of the assay manufacturer (Roche Diagnostics, Mannheim, Germany). NT-proBNP was analyzed using the log transformation as the clinical variance then becomes normally distributed [15,16], and recalculated for presenting cut-off values.

Echocardiography: After transthoracic echocardiography (TTE), all patients received transesophageal echocardiography (TEE) using either a Philips iE33 or Epiq echocardiographic device (Philips, Hamburg, Germany). Vegetation was defined as a definite floating structure, which is irregular in character and is located on the surface of the specific valve. Vegetation size was measured in the longest axis presenting. An abscess was diagnosed when a definite region of reduced echo density was found within the valvular annulus or adjacent myocardial structures were found in the setting of valvular infection.

F-18-FDG PET/CT: A high focal uptake of F-18-FDG shows the active infectious process. The scans were performed on a Siemens Biograph 16 (CTI-Siemens, Erlangen, Germany) with 16-slices CT multidetector (0.5 s/revolution) or on a Biograph mCT40 flow (CTI-Siemens, Erlangen, Germany) with 40-slices CT-multidetector (0.5 s/revolution). The standard scan was made from skull to upper thigh with elevated arms, 3 min per bed position. The low-dose CT was performed with 50 mAs and 120 kVp. No contrast-enhanced CT scans were performed. We used standard reconstruction parameters with a 5 mm slice thickness with vendor-recommended reconstruction parameters. Images were analyzed using the software syngo.via (Siemens, Erlangen, Germany). An F-18-FDG PET/CT scan was preferably conducted if echocardiographic image quality was suboptimal, e.g., in the presence of prosthetic valves.

Microbiological examination: All valve cultures and valve polymerase chain reaction (PCR) tests were conducted at the local medical center. Blood samples of every patient were cultured. In 87 cases, blood cultures were performed by referring hospitals. Local blood cultures were processed in the BD Bactec FX system (Becton and Dickinson, Heidelberg, Germany). Susceptibility testing was performed according to the guidelines. For valve PCR, molecular species identification was carried out by applying broad-range PCR amplification methods accredited according to DIN EN ISO 15189. Bacterial V7-9 variable regions of the 16S ribosomal gene and fungal 18S rRNA gene regions were amplified from total nucleic extracts followed by sanger-based DNA sequencing on ABI PRISM^®^ 310 Genetic Analyzer. DNA sequences were further analyzed with the Integrated Database Network System SmartGene centroid database.

Tissue preparation and histopathological examination: After surgical removal, specimens were fixed in buffered formalin solution (3.7%) overnight at room temperature. Specimens were then processed with a view to dehydration and paraffin embedding by use of standardized and automated techniques (Shandon Pathcentre, Thermo Electron Corporation, Waltham, MA, USA). Histological sections of 5 µm thickness were performed, deparaffinized and stained with hematoxylin/eosin in a fully automated system (Histostainer Plus, Dako, Hamburg, Germany). The histological criteria for infectious endocarditis were given by a fibrin-thrombus with granulocytic infiltration and bacteria colonies with or without ulceration of the endocard.

Endpoint definition of (a) infective endocarditis: All patients were treated surgically due to a “definite” diagnosis according to the modified Duke criteria [13]. For this study, the final diagnosis was proven (or rejected) by a clinical committee consisting of experienced physicians from the specialties of cardiology, heart surgery, microbiology and histopathology based on all available clinical information (surgery report, histopathological findings, imaging, laboratory results, microbiological tests, discharge letter). A decision was made by consensus in all cases. (b) mortality: if not interpretable from available information, vital status was acquired by a telephone follow-up.

Statistical Analysis: Qualitative data were expressed as frequencies, and quantitative data as mean ± SD. Comparison between groups was made using Fisher‘s exact test for categorical variables and Student’s t-test for continuous variables. For evaluation of the predictive power of the outcomes (a) diagnosis of IE, (b) mortality after 30 days and (c) mortality after 1 year of follow-up, simple (and, if needed, multivariate) regression analysis was performed (statistical software: Graphpad Prism 9, San Diego, CA, USA). The cut-off point for optimal sensitivity/specificity was estimated by the Youden-index [17]. A *p* value <0.05 was considered significant.

## 3. Results

### 3.1. Study Population

Two hundred fifteen consecutive patients (mean age 64 ± 13 years, 74% male) with surgical therapy for left-sided IE were analyzed. Among these patients, 201 (93.5%) were finally diagnosed with IE and, in 14 patients (6.5%), the diagnosis of IE was rejected. The aortic valve was affected in 135 patients (62.8%), the mitral valve in 101 (47.0%) and both in 21 (9.8%). Poor left-ventricular function was present in 19 patients (8.8%), and the mean logistic EuroSCORE II was 15.6 ± 10.9%. Septic embolisms occurred mainly cerebrally and renally, all together in 151 patients (70.2%); see Table 1 for baseline characteristics.

### 3.2. Examination of Qualitative Parameters

**Figure 1:**
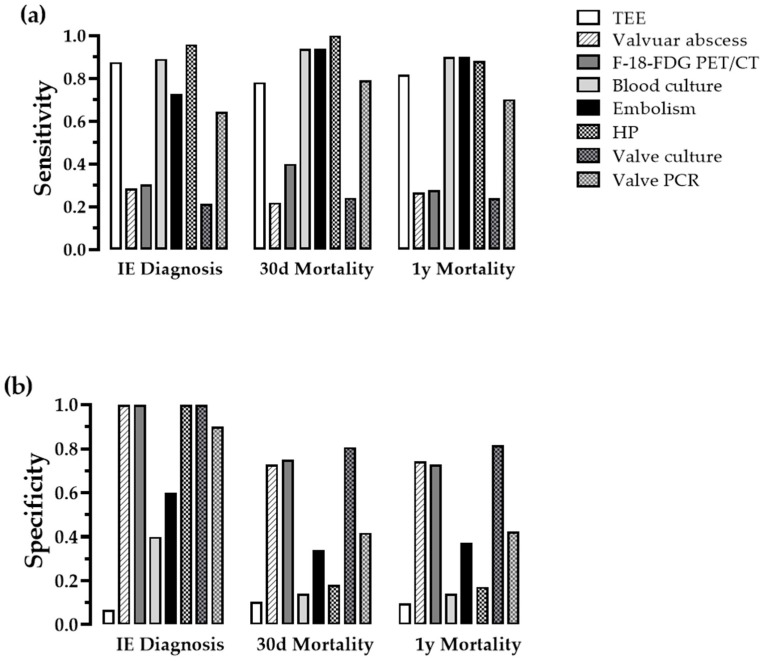
Contingency analysis of qualitative diagnostic tools. Sensitivity (**a**) and specificity (**b**) for IE diagnosis, 30-day and 1-year mortality of imaging, microbiologic, nuclear and histopathological diagnostics. HP = histopathology; other abbreviations as Table 2.

Eight qualitative parameters were analyzed, and three of them (HP, valve culture, valve PCR) were collected perioperatively (Table 2). The highest sensitivity for the diagnosis of IE had TEE, blood cultures and HP. The highest specificity had F-18-FDG PET/CT, the detection of a valvular abscess (via TEE or computed tomography), HP and valve culture (Figure 1). The highest sensitivity for the outcome 30-day mortality and 1-year mortality had blood cultures, detection of systemic embolism, HP, valve PCR and TEE, whereas valve culture, F-18-FDG PET/CT, and the absence of valvular abscess bore the highest specificity. Despite its intermediate sensitivity, valve PCR was beneficial for detecting germs like Cutibacterium acnes and Granulicatella sp. in blood culture-negative patients.

### 3.3. Prediction of IE by Quantitative Parameters

**Figure 2:**
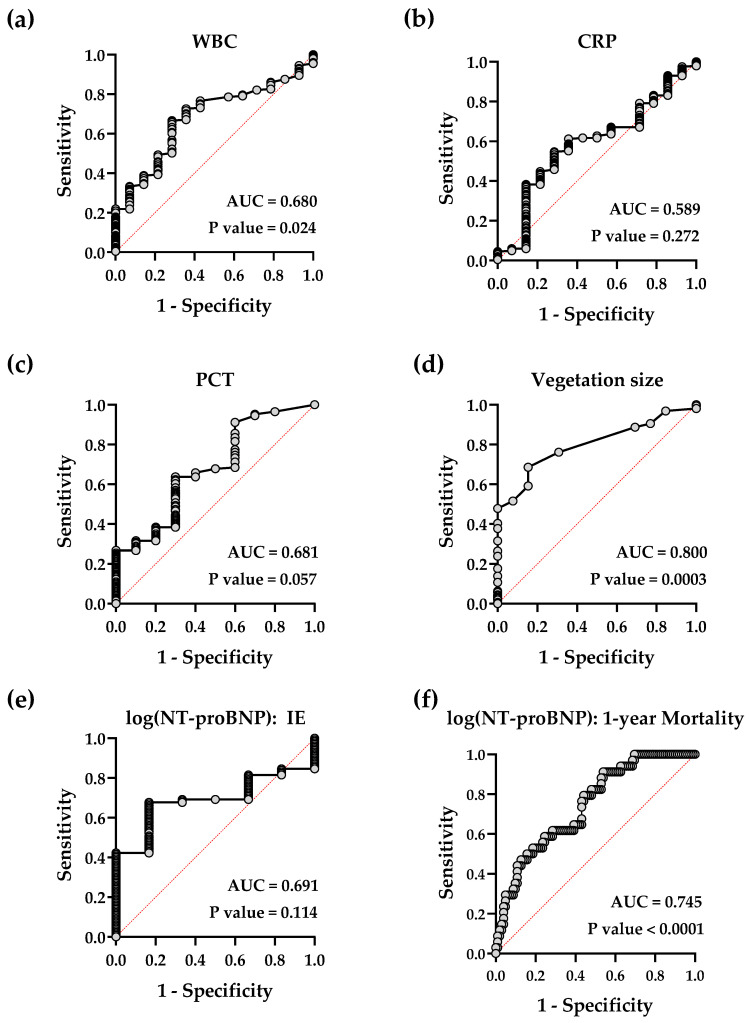
Simple logistic regression of quantitative diagnostic tools. Receiver-operating characteristic curves showing the predictive power for diagnosis of either IE (**a**–**e**) of white blood cell count (WBC), C-reactive protein (CRP), procalcitonin (PCT), vegetation size and N-terminal prohormone of B-type natriuretic peptide (NT-proBNP) or 1-year mortality of NT-proBNP (**f**).

White blood cell count (WBC) and the inflammatory marker proteins CRP and PCT had a moderate power for prediction of IE, with WBC being the only one of significant power (AUC 0.680, *p* value 0.024) in ROC analysis (Figure 2a–c, Table 3).

The highest AUC was reached by the measurement of the vegetation size via TEE with an AUC = 0.800 (*p* value 0.0003, Figure 2d). The optimal cut-off value was a vegetation size of 11.5 mm (Table 3), a sensitivity of >90% was reached by a vegetation size of <8.48 mm, and a specificity of 90% by size of >13.49 mm.

**Figure 3:**
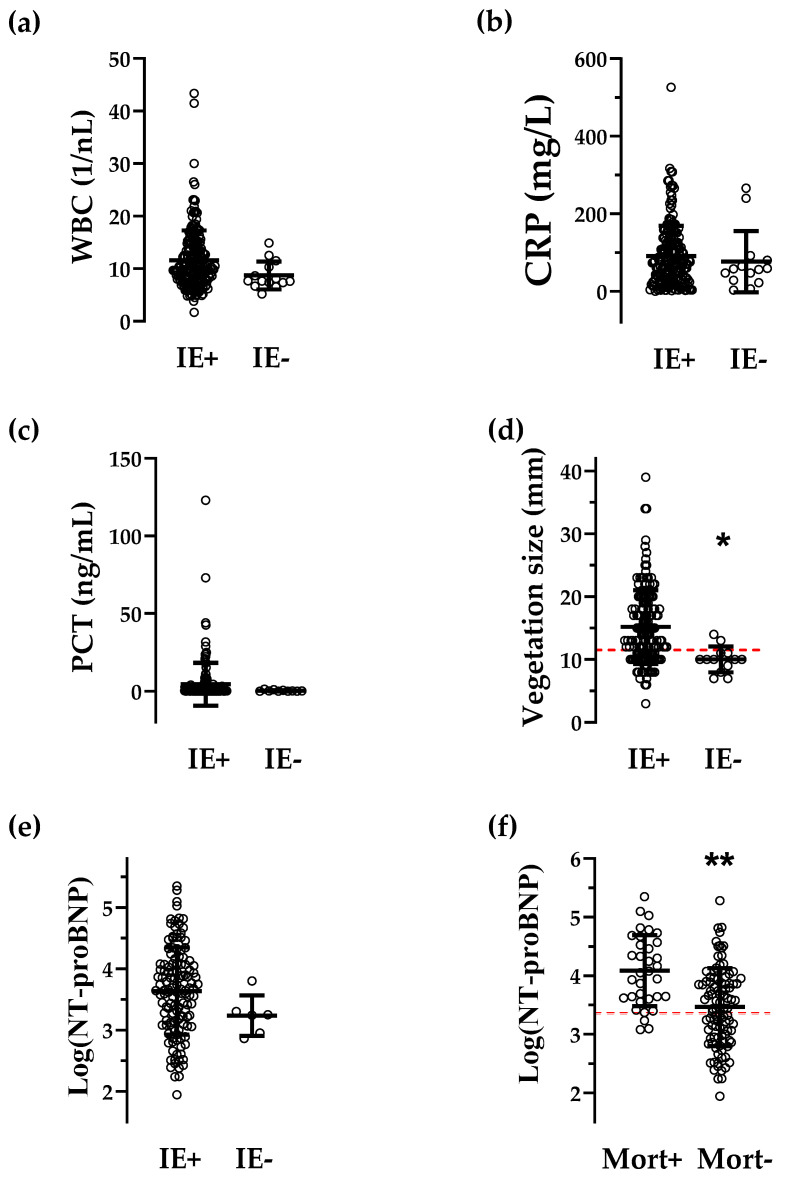
Intergroup differences for quantitative parameters. Scatter plots of serum markers for inflammation (WBC, CRP and PCT, **a**–**c**), of a marker for cardiac stress (NT-proBNP, (**d**) and of the vegetation size (measured by transesophageal echocardiography, (**e**) in patients who underwent operative therapy for IE with confirmed (IE+) or rejected (IE-) diagnoses for IE. (**f**): Patients with positive (Mort+) vs. negative mortality (Mort-) after 1 year of follow-up. Red dotted lines represent the optimal cut-off values for vegetation size (**d**) and log(NT-proBNP) (**f**), comparison of Table 3 and Table 4b. Bars represent the mean ± SD. * *p* < 0.01, ** *p* <0.0001, Student’s t-test. Abbreviations as in Table 1.

The cardiac stress marker NT-proBNP failed to be of predictive power for the detection of IE but was highly significant in predicting mortality with an odds ratio (95%-CI) of 4.70 (0.72, 2.50) for the 30-day mortality and of 4.33 (2.2, 9.1) for 1-year mortality (Figure 2e,f, Table 3 and Table 4). Inflammatory markers were not significantly different between patients with and without IE, with WBC closely missing significance level (*p* = 0.058; Figure 3a–c).

Vegetation size was smaller in IE-negative patients (Figure 3d). Of the 14 patients being IE-negative, in 10 cases, diagnoses would have been rejected if the novel calculated cut-off for vegetation size (11.5 mm, Figure 3c) had been requested for diagnosis. The remaining four patients were below the cut-off values for WBC and CRP, and thus the IE diagnosis could have been rejected if the presence of the novel calculated cut-off values for these inflammatory parameters (see Table 3) had also been required. Supplemental Table S1 shows the diagnosis and characteristics of these 14 patients. As for IE prediction, vegetation size confirmed its predictive power in multivariate regression analysis (Table 3). NT-proBNP levels showed no difference between IE-positive and -negative patients (Figure 3e).

### 3.4. Prediction of Mortality

Log(NT-proBNP) was higher in the group of patients with positive 1-year mortality (Figure 3f) and showed a high predictive power for 30-day (AUC 0.750, *p* value < 0.001) as well as 1-year mortality (AUC 0.745; *p* value < 0.0001, Table 4). The cut-off values of NT-proBNP for 30-day and 1-year mortality were 3990.2 pg/mL and 2322.7 pg/mL. CRP had an acceptable predictive power for 30-day mortality (Table 4), which declined for the 1-year mortality (Table 4). Other parameters (WBC, PCT and vegetation size) did not have conclusive AUCs (values < 0.7).

**Figure 4:**
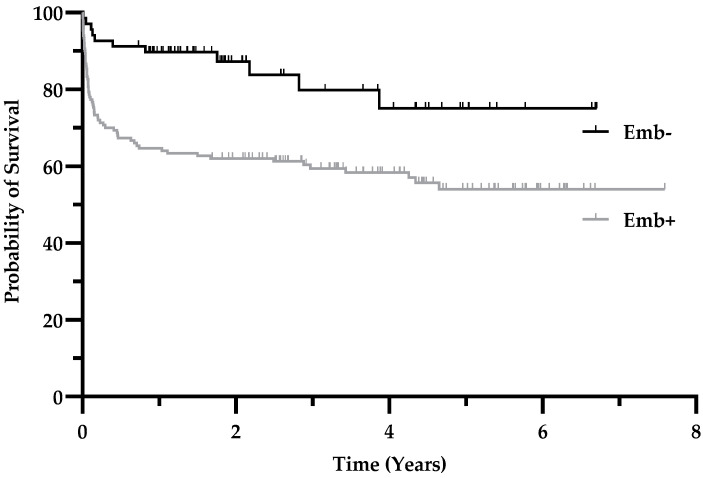
Presence of systemic embolism reduces the probability of survival. Kaplan–Meier estimates showing the probability of survival of patients with IE and surgical treatment and who had systemic embolism (Emb+) compared to patients without embolism (Emb-). The *p* value for the intergroup difference is < 0.001; see text for details.

As mentioned earlier, the presence of embolism had a high sensitivity for mortality with the highest value (0.94) amongst other preoperative diagnostic tools for the 30-day mortality (Figure 1a). To confirm this observation, we calculated survival proportions of patients with embolic events vs. patients without embolic events and plotted them over time. Figure 4 shows survival curves for these populations. The curves are significantly different in the log-rank (Mantel–Cox) test (p = 0.0007) and in the Gehan–Breslow–Wilcoxon test (p = 0.0003) with a 95% confidence interval of 1.83–4.87 (logrank). At 2 years of follow-up, the survival rate was 87.3% for patients without embolism vs. 62.0% with embolism. The relative risk for 1-year mortality in patients with embolism (vs. without) is 3.4 (95% CI 1.6–7.1, p = 0.001 with a number needed to treat (harm) of 4.04).

## 4. Discussion

### 4.1. Microbiological Testing Is a Mainstay in Diagnosing IE

The sensitivity and specificity of blood cultures with their respective microorganisms have been reported many times. In our cohort, the identification of causative agents was possible in 89% of patients, which is consistent with previous studies [18,19,20,21]. Valve culture showed a minor sensitivity compared to valve histopathology, and there are only a few studies that have addressed this important issue [22,23]. Munoz et al. reported a sensitivity of 25% for valve cultures in 71 patients, whilst Greub et al. reported 13% in 127 patients. In our study, we could observe a sensitivity of 21% for valve cultures and 64% for valve PCR, suggesting that many patients received efficient antibiotic therapy. This is consistent with a landmark study demonstrating that valve culture results depend on the length of preoperative antibiotic treatment [24]. Despite the low sensitivity of valve culture and PCR, microbiological analysis of valve specimens remains important to detect pathogens such as *Tropheryma whipplei* or *Coxiella burnetii,* which cannot be detected by routine culture-based diagnostics [25], in order to guide antibiotic therapy.

### 4.2. TEE and Establishment of a Cut-off Value for Vegetation Size

Primarily used as a screening method following transthoracic echocardiography, TEE not only provides information about the extent of valve infection but also about the involvement of the perivalvular area and allows quantification of the vegetation size. These criteria are crucial and may immediately promote operative therapy following guidelines [7,8].

In our study, TEE showed poor specificity (Table 2, Figure 2). The distinction between infectious vs. degenerative changes in valve morphology obviously remains challenging. In many cases, the clinical situation, which is primarily represented by the Duke criteria, guides the interpretation of these valve structures. We correlated the size of the vegetation with the final IE diagnosis with regression analysis. The AUC for vegetation size showed good power; further, we calculated a novel cut-off value for an optimal sensitivity/specificity, which was 11.5 mm, with higher values favoring IE diagnosis. Application of the cut-off values for vegetation size and the inflammatory markers WBC and CRP would have ruled out the diagnosis of IE in the 14 IE-negative surgically treated patients. However, nine patients had aortic regurgitation ≥ 2, six had mitral regurgitation ≥ 2, and two patients had both; therefore, there were indications for surgery. These results confirm the popular threshold size for vegetations of 10 mm [7,9,26], possibly because of the fact that smaller “vegetations” turn out as degenerative alterations when inspected during surgery and analyzed by histopathology and microbiological testing.

### 4.3. Patient Population and Affected Valves

Compared to the literature, the actual collective is slightly older than IE patients in previous decades. An epidemiologic study shows an increasing mean age of the patients in the last decades from 45 years in the 1980s up to 55–60 years in 2000 [27]. This study also demonstrates increasing the male gender by up to 75% from 1970 to 2000. However, the age of patients with IE in recent studies was as high as 63 ± 15 (mean ± SD) years [28] and 64 (52–73) years (mean, interquartile range) [29] and thus comparable to the population our study (64 ± 13 years). The aortic valve was affected about 1.5 times more often than the mitral valve, which lies within the wide range of studies in the literature, which varies from a two-fold dominance of the mitral over the aortic valve or vice versa [2,29,30]. At the same time, the reported combined affection of aortic and mitral valves constantly ranges around 10% [2,3,14,29], like in the actual study. Surgically treated patients in the literature had severe valve regurgitations between 30% and 87% [21,31]. We found moderate to severe regurgitations in around 60% of the patients. Prosthetic valves were used in 25% of our study population, which is in the range of previous studies (21% to 28%) [3,21,29]. These valves regularly deflect the echo beam leading to reduced sensitivity of echocardiography for the detection of IE. F-18-FDG PET/CT is known to have a high sensitivity and specificity, which may vary within patient characteristics and is especially useful in patients whose prosthetic valves had been implanted >3 months ago [7,12,32,33].

In our study, patients with impaired echocardiographic image quality due to a prosthetic valve preferably received an F-18-FDG PET/CT scan. The high specificity observed in this study makes this an excellent method complementary to echocardiography.

### 4.4. Nt-Probnp Had Predictive Power for Mortality, Presence of Systemic Embolism Was Associated with Mortality

Recently, it has been shown that NT-proBNP levels below 2926 pg/mL had a favorable in-hospital outcome in patients with acute IE 9]. In our analysis, NT-proBNP had predictive power for the 30-day and the 1-year mortality with values < 3990 pg/mL and <2323 pg/mL favoring survival. Vegetation size failed to do so, although clearly associated with predictive power for the diagnosis of IE. The latter could somehow be surprising at first glance, but it is in line with a large study comparing surgical vs. medical-only therapy in patients with a vegetation size of <10 mm vs. >10 mm: Fosbol et al. showed that in 1006 patients with left-sided IE, medically managed patients with a vegetation size of >10 mm had a lower probability of survival, whereas surgically treated patients were indifferent [9].

In our study, embolic events were relatively common, with an event rate of 70%. In the literature, the overall risk of peripheral embolism is described as between 20–50%, and cerebral embolism occurs in up to 60% of IE patients [34]. A meta-analysis could correlate the embolic risk with vegetation size [26]. Our data show an increased risk for mortality in the presence of systemic embolism, which was not associated with vegetation size. This discrepancy can also be explained by the fact that, in our study, only operated patients were examined. Notably, the Kaplan–Meier survival curve for the cohort with systemic embolism parallels after approximately 1 year (please compare Figure 4), which may lead to false negative estimates of shorter follow-ups if outcomes of patients with embolism are analyzed.

### 4.5. Study Limitations

This study recruited retrospectively; thus, not every patient had the entire palette of diagnostic tests. Further, we found that there were 10 HP-negative patients in our collective who nonetheless had positive blood cultures with pathogens that were compatible with an IE. In four of these patients, the duration of preoperative antibiotic treatment was >20 days. Thus, the affected valve may have healed by the time of surgery. An alternative site of infection could be identified in those patients (Supplemental Table S1). In three patients, blood culture results were of questionable relevance and were counted positive for analysis; if counted negative, there would be no significant change in sensitivity/specificity. Once surgery is performed, HP can be very helpful in guiding postoperative therapy, especially when blood cultures, valve cultures and valve PCR remain negative or when the microorganism that has been isolated is a contaminant [35]. Another limitation is the spare use of F-18-FDG PET/CT scans, which were reserved for patients with ambiguous diagnostic results and/or suboptimal echocardiographic imaging. Thus, F-18-FDG PET/CT results have to be interpreted with caution.

## 5. Conclusions

In addition to previous knowledge and utilization of the cut-off value for vegetation size, we could add information that a vegetation size below our calculated cut-off value of 11.5 mm unlikely favors endocarditis. In combination with the cut-off values for inflammatory markers, clinicians have a gain in diagnostic arguments for decision-making. In terms of mortality, the presence of systemic embolism showed a significant association, which argues in favor of early surgical therapy. NT-proBNP, as an indicator of mortality, can help to identify patients at risk in order to recommend and utilize a tighter follow-up scheme.

## Figures and Tables

**Table 1:** Baseline characteristics.

Parameter	*n* = 215
Age (years)	64.2 ± 13.3
Male	160 (74.4)
BMI	28.2 ± 11.9
Logistic EuroSCORE II	15.6 ± 10.9
Coronary artery disease	50 (23.2)
Peripheral occlusive disease	28 (13.0)
Diabetes mellitus	41 (19.1)
Malignancies	31 (14.4)
Serum kreatinin (mg/dL)	1.6 ± 2.2
Chronic hemodialysis	18 (8.4)
Immunosuppression	15 (7.0)
i.v. drug abuse	9 (4.2)
NYHA functional class III/IV	104 (48.4)
Native valves	160 (74.4)
IE affected valves	
Aortic valve only	114 (52.3)
Mitral valve only	80 (37.2)
Combined aortic and mitral	21 (9.8)
Regurgitation degree II/III	
Aortic valve	78 (57.7)
Mitral valve	68 (67.3)
LVEF > 50%	142 (66.0)
LVEF < 30%	19 (8.8)
Embolism	151 (70.2)
Cerebral	77 (35.8)
Renal	38 (17.7)
Spleen	21 (9.8)
Eyes	13 (6.0)
Other	10 (4.6)
Fever	185 (86.0)
WBC (1/nL)	11.4 ± 5.5
CRP (mg/L)	90.1 ± 77.9
PCT (ng/mL)	4.3 ± 13.3
Vegetation size (mm)	14.8 ± 5.8
NT-proBNP (pg/mL)	4398 (1310, 11,830)
Log(NT-proBNP)	3.62 ± 0.70

Values are n (%) or mean ± SD, except for NT-proBNP with median (IQR). BMI = body mass index; CRP = C-reactive protein; IE = infective endocarditis; LVEF = left ventricular ejection fraction; NT-proBNP = N-terminal prohormone of B-type natriuretic peptide; NYHA = New York Heart Association; PCT = procalcitonin; WBC = white blood cell count.

**Table 2:** Frequencies of qualitative diagnostic tests.

	IE Diagnosis			Mortality 30-Days			Mortality 1-Year		
Parameter	Pos.	Neg.	*p*	Pos.	Neg.	*p*	Pos.	Neg.	*p*
**TEE**			>0.999			0.079			0.102
pos.	175	14		25	164		49	140	
neg.	25	1		7	19		11	15	
**Abscess**			**0.013**			0.665			>0.999
pos.	57	0		7	50		16	40	
neg.	143	15		25	133		44	115	
**PET/CT**			0.176			0.440			>0.999
pos.	17	0		4	13		5	12	
neg.	39	6		6	39		13	32	
**Blood culture**			**0.006**			0.269			0.503
pos.	178	9		30	157		54	133	
neg.	22	6		2	26		6	22	
**Embolism**			**0.016**			**0.001**			**<0.001**
pos.	145	6		30	121		54	97	
neg.	55	9		2	62		6	58	
**Histopathology**			**<0.001**			0.121			0.759
pos.	94	0		14	81		22	72	
neg.	4	14		0	18		3	15	
**Valve culture**			0.129			0.613			0.420
pos.	37	0		7	30		13	24	
neg.	137	11		22	126		41	107	
**Valve PCR**			**0.001**			0.070			0.161
pos.	103	1		19	85		33	71	
neg.	57	9		5	61	10	14	52	

Distribution of frequencies for diagnosis of IE, 30-day and 1-year mortality. P = *p* value; PCR = polymerase chain reaction; PET/CT = F-18-FDG PET/CT; TEE = transesophageal echocardiography. Fischer’s exact test was applied, a *p* value < 0.05 was considered significant. Bold font highlights the respective diagnostic method and its *p* value, if significant.

**Table 3:** Characterization of quantitative parameters for IE.

**Simple logistic regression analysis on outcome IE.**							
**Parameter**	**Cut-off**	**Sens.**	**Spec.**	**80% Sens.**	**80% Spec.**	**AUC**	***p*** **Value**
**WBC (1/nL)**	8.75	0.67	0.73	7.34	11.55	0.680	0.024
**CRP (mg/L)**	66.10	0.57	0.73	28.72	93.67	0.589	0.272
**PCT (ng/mL)**	0.50	0.60	0.64	0.41	1.22	0.681	0.057
**Vegetation size (mm)**	11.50	0.69	0.86	9.49	11.50	**0.800**	**<0.001**
**log(NT-proBNP)**	3.37	0.68	0.83	3.06	3.37	0.691	0.114
**Multivariate logistic regression analysis on outcome IE.**							
**Parameter**		**OR (95%-CI)**		***p*** **Value**		**C Index**	
**WBC (1/nL)**		1.37 (0.98, 2.16)		0.110			
**CRP (mg/L)**		1.00 (0.98, 1.03)		0.707		0.909	
**Vegetation size (mm)**		**1.58 (1.12, 2.64)**		**0.037**			
**log(NT-proBNP)**		1.91 (0.41–12.83)		0.449			

Analysis of PCT was omitted in the multivariate analysis because of collinearity. AUC = area under the curve; CI = confidence interval; Sens. = sensitivity; Spec. = specificity; other abbreviations as in Table 1. Bold font highlights the quantitative parameter and the respective test result, if significant.

**Table 4:** Characterization of quantitative parameters for mortality.

Simple logistic regression analysis on outcome 30-day mortality.							
**Parameter**	**Cut-off**	**Sens.**	**Spec.**	**80% Sens.**	**80% Spec.**	**AUC**	***p*** **Value**
**WBC (1/nL)**	11.27	0.50	0.62	7.71	14.85	0.543	0.430
**CRP (mg/L)**	76.60	0.85	0.58	77.66	138.69	**0.719**	**<0.001**
**PCT (ng/mL)**	1.63	0.54	0.74	0.21	2.43	0.624	0.046
**Vegetation size (mm)**	10.53	0.83	0.28	10.53	19.50	0.553	0.413
**log(NT-proBNP)**	3.61	0.88	0.52	3.61	4.08	**0.750**	**<0.001**
Simple logistic regression analysis on outcome 1-year mortality.							
**Parameter**	**Cut-off**	**Sen.s**	**Spec.**	**80% Sens.**	**80% Spec.**	**AUC**	***p*** **Value**
**WBC (1/nL)**	12.51	0.42	0.72	7.75	14.89	0.547	0.278
**CRP (mg/L)**	59.78	0.77	0.55	49.76	142.91	0.620	0.006
**PCT (ng/mL)**	1.21	0.72	0.49	0.81	2.03	0.627	0.012
**Vegetation size (mm)**	16.50	0.44	0.67	8.51	19.50	0.552	0.296
**log(NT-proBNP)**	3.37	0.92	0.45	3.60	4.04	**0.745**	**<0.0001**

Abbreviations as in Table 3. Bold font highlights the quantitative parameters and the respective test result, if significant.

## Data Availability

The data presented in this study are available from the corresponding author upon reasonable request.

## Supplementary Materials

The following supporting information can be downloaded at: https://www.mdpi.com/article/10.3390/jcdd9120467/s1, Table S1: Characteristics of IE-negative patients.
