# Supplementary material for: Infective Endocarditis: Predictive Factors for Diagnosis and Mortality in Surgically Treated Patients
Source: J Cardiovasc Dev Dis. 2022 Dec 19;9(12):467. doi: 10.3390/jcdd9120467 (PMC9788195; doi:10.3390/jcdd9120467)
Supplement: Supplementary file 1 [file jcdd-09-00467-s001.zip › jcdd-2007631-supplementary.pdf]

Supplemental Table S1: Characteristics of IE-negative patients

Pat	CRP (mg/L)	PCT (ng/mL)	WBC (1/nL)	Veg. Size (mm)	Blood culture	Valve cult	Valve PCR	Valve	Intraoperative finding and histopathology	Alt. diagnosis
1	2.9	0.01	7.7	13	Staphylococcus hominis	Neg.	Neg.	Aortic	Degenerative	Pneumonia
2	22.0	0.20	14.9	10	Negative	Neg.	Neg.	Mitral	Perforation (degenerative)	Cardiac decompensation with pneumonia
3	6.2	n.d.	6.7	14	Negative	Neg.	Neg.	Aortic	Degenerative	Pneumonia
4	80.0	n.d.	10.3	7	Streptococcus pneumoniae	Neg.	Neg.	Mitral	Perforation	Cardiac decompensation with pneumonia
5	65.0	n.d.	12.5	11	Streptococcus salivarius	Neg.	Neg.	Aortic	Degenerative	Urosepsis with dialysis
6	59.0	0.18	7.6	10	MSSA	Neg.	Neg.	Aortic	Degenerative	Pneumonia
7	92.0	0.01	6.6	8	Enterococcus faecalis	Neg.	Neg.	Mitral	Degenerative	Colon cancer
8	47.0	1.31	7.3	10	MSSA	Neg.	Neg.	Mitral	Degenerative	Pneumonia
9	56.0	0.65	8.7	12	Streptococcus oralis	Neg.	Neg.	Aortic	Degenerative	Cardiac decompensation with pneumonia
10	266.0	0.17	11.5	10	Negative	Neg.	Neg.	Mitral	Degenerative	Sepsis
11	240.0	0.99	8.1	10	MSSA	Neg.	Neg.	Mitral	Excessive calcification	Urosepsis with dialysis
12	58.0	0.05	7.4	10	Negative	Neg.	Neg.	Mitral	Degenerative	Cardiac decompensation with pneumonia
13	28.7	0.06	7.7	7	Negative	Neg.	Neg.	Aortic	Degenerative	Cardiac decompensation
14	47.0	n.d.	5.2	12	Streptococcus viridans	Neg.	Neg.	Aortic	Degenerative	Acute pericarditis

In these 14 patients, IE diagnosis was rejected mainly based on perioperative diagnostics. Abbreviations: CRP = C-reactive protein; IE = infective endocarditis; MSSA = methicillin-susceptible staphylococcus aureus; Neg. = negative, PCT = procalcitonin; PCR = polymerase chain reaction; Veg. = vegetation; WBC = White blood cell count.
